# Use of Physical and Intellectual Activities and Socialization in the Management of Cognitive Decline of Aging and in Dementia: A Review

**DOI:** 10.1155/2012/384875

**Published:** 2012-12-31

**Authors:** Myuri Ruthirakuhan, Angela C. Luedke, Angela Tam, Ankita Goel, Ayaz Kurji, Angeles Garcia

**Affiliations:** Department of Medicine (Geriatrics) and Centre for Neuroscience, Queen's University, Kingston, ON, Canada K7L 5A2

## Abstract

Lifestyle nonpharmacological interventions can have a deep effect on cognitive aging. We have reviewed the available literature on the effectiveness of physical activity, intellectual stimulation, and socialization on the incidence of dementia and on the course of dementia itself. Even though physical activity appears to be beneficial in both delaying dementia onset and in the course of the disease, more research is needed before intellectual stimulation and socialization can be considered as treatments and prevention of the disease. Through our paper, we found that all three nonpharmacological treatments provide benefits to cognition and overall well-being in patients with age-related cognitive impairments. These interventions may be beneficial in the management of dementia.

## 1. Introduction

Alzheimer's disease (AD) is a neurodegenerative disorder with devastating consequences [1]. Despite being the most common cause of dementia, affecting approximately 5.4 million Americans [2] and almost 50% of people over the age 85 [3], no cure has yet been discovered. Efforts are also focusing on the development of more effective strategies to slow the progression of AD to increase the quality of life of those affected. Even a two-year delay in disease onset would reduce the prevalence of AD among Americans by two million people within fifty years [4]. If an intervention that delayed the onset of AD by five years had been applied back in 1997, we would have seen a 50% reduction in AD incidence [4]. Research on strategies to slow the development and progression of AD is arguably more important now than ever before, since the number of people with AD is expected to nearly triple over the next forty years [4], and dementia is the most important contributor to disability in the elderly [5].

Among others, three nonpharmacological interventions are particularly relevant as they might positively influence cognition, general functioning, and overall quality of life. These three strategies are *physical exercise*, *intellectual stimulation,* and *social interaction*. While there are studies that evaluate the role of individual and multimodal interventions on AD, there is a lack of literature on the combination of all three. The purpose of this paper is to review key areas of the literature that focus on the effects of physical exercise, intellectual stimulation, and socialization strategies on AD evolution, as they collectively play an important role in the management of Alzheimer's disease. Physical exercise encapsulates both aerobic exercises (e.g., walking and cycling) and nonaerobic exercises (e.g., strength and resistance training; flexibility and balance exercises). For intellectual stimulation, we examine studies that have evaluated the prognostic effects of either cognitive hobbies (e.g., reading, word puzzles, and card games) or cognitive training (e.g., computer training games/paradigms that target specific cognitive domains such as memory and attention). Social interaction is defined as the participation of an AD patient in group-related activities, such as mealtime conversations, support groups, or other forms of social engagement.

Evidence-based nonpharmacological interventions are an essential part of any management plan, especially for geriatric populations in whom the harmful effects of polypharmacy are a major concern. Nonpharmacological strategies are sometimes even more effective than pharmacological interventions and are empowering to proactive individuals who seek to gain greater personal control over their mental and physical health. We suggest that comprehensive AD management plans should include evidence-based nonpharmacological strategies.

## 2. Methods

Electronic databases (PubMed, Web of Science, and MEDLINE) were searched for the period from 1980 to 2012 with the combination of key words: AD, dementia, incident AD, aging, cognition, cognitive aging, exercise, aerobic exercise, resistance training, cognitive reserve, intellectual stimulation, cognitive stimulation, hobbies, cognitive training, memory training, brain games, leisure activities, socialization, loneliness, social network, support groups, quality of life, activities of daily living, nonpharmacological, and treatment. We included studies that examined the preservation of cognition in AD populations and AD incidence.

## 3. Physical Activity 

The health benefits attributed to physical activity are numerous and well known. Exercise has been associated with a lower incidence in many chronic diseases, such as coronary heart disease [6], type 2 diabetes [7], obesity [8], cancer [9], bone loss [10], and high blood pressure [11]. We have reviewed the effects of physical exercise on cognition.

Higher cardiorespiratory fitness has been related to higher scores on tests of cognitive function [12]. A meta-analysis of randomized controlled trials examining the relationship between exercise and cognition showed modest improvements in attention, processing speed, executive function, and memory among older adults in the treatment arms [13]. This is highly relevant for the elderly population, as it suggests that physical activity can serve as a preventative measure against age-related cognitive decline [14]. 

Several large longitudinal studies followed older adults without cognitive impairments at baseline and measured rate of incident dementia to clarify the relationship between physical activity and incident cognitive loss. A large prospective study by Podewils et al. identified an inverse relationship between physical activity and dementia risk [15]. Compared to no exercise, physical activity has been linked with reduced risks of developing cognitive impairment and dementia [16] with the risk for dementia being further reduced with increasing levels of physical activity. Larson and colleagues found that persons who exercised three or more times per week had a reduced risk of developing dementia compared to those who exercised less, and the reduction was more marked among those with the poorest physical function at baseline [17]. These results were corroborated by Buchman et al. who found that participants in the lowest percentiles of physical activity had more than twice the risk of developing dementia than those in the highest percentiles of physical activity [18]. Furthermore, Lautenschlager et al. demonstrated that these results might be transferable to adults with mild cognitive impairment (MCI), and, thus, at high risk for dementia; participants who underwent exercise training showed modest improvements in cognition after six months [19]. Physical exercise has, therefore, been recommended as a preventative measure of mild cognitive impairment and dementia [20, 21]. 

There is much less research focusing on the effect of physical activity in AD patients. This may be due to the challenges of implementing an exercise regime while managing the behavioral and emotional disturbances in AD patients, particularly in the later stages of the disease. However, the results in the available literature are promising. Early research involving AD patients in nonrandomized controlled trials showed significant cognitive improvements among participants who underwent cycling training and somatic and isotonic-relaxation exercises [22, 23]. 

Physical exercise may have beneficial effects in AD patients beyond cognition as well. A meta-analysis on 30 randomized controlled trials that employed exercise, behavioral and environmental manipulations in patients with cognitive impairment found exercise had positive effects on strength and cardiovascular fitness in addition to improvements in behavior and cognition [24–26]. Further evidence supporting multifaceted positive effects of exercise on AD can be traced to recent randomized controlled trials of physical exercise regimes on AD patients (Table 1).

Compared to controls, patients in the intervention programs showed better physical functioning (functional reach, walking, and mobility). After treatment, these patients also showed improved performance of activities of daily living (ADLs), and less cognitive decline and cognitive improvement in some cases. Physical exercise, therefore, appears to be beneficial for AD patients. While the majority of the studies did not find any differences in depression, one study by Steinberg found increased depression and decreased quality of life in patients who underwent the exercise intervention [31]. Further research into the effect of physical exercise on mood and quality of life in AD patients is, therefore, required. 

When considering the role of exercise on AD, it is important to note that any positive results may be due to a placebo effect, even in randomized controlled trials. However, due to the varied nature of the outcome measures used in these studies, it is unlikely that every intervention group demonstrated significant gains over the controls due to a placebo effect alone. Furthermore, control group members never appeared to show any improvement and often showed higher rates of functional and cognitive decline. 

The majority of the reviewed studies employed aerobic exercise as a component to their therapy. Yet, two studies demonstrated that nonaerobic activity, such as strength and flexibility training, might also be beneficial to AD patients [29, 34]. Studies involving cognitively normal adults and nonaerobic exercises offer insight into the potential effects of nonaerobic activity and cognitive decline. Cassilhas et al. found that participants who underwent resistance training showed significant improvements in memory measures compared to controls [35]. Liu-Ambrose and colleagues, who used a combination of resistance and balance exercises as their intervention, saw significant improvements in response inhibition in their intervention group while the control group deteriorated [36]. Resistance training may, therefore, provide protective effects for cognition. The effect of stretch exercises, such as yoga, on cognition has been briefly examined; Oken and colleagues found no cognitive differences between their control and yoga groups after treatment [37]. However, more research into stretch exercises and cognition is required to draw more definitive conclusions. Future studies may investigate which types of exercises, or combinations of exercises, yield the greatest benefit specifically to AD patients. It is important to mention that some of the aforementioned randomized controlled trials [27, 29, 30, 32] were conducted on participants recruited from long-term care facilities, and thus were likely to be in the more moderate-advanced stages of the disease. These studies demonstrate not only the feasibility of the exercise programs in AD patients but also that patients can benefit greatly from physical exercise even in moderate-to-severe stages of the disease. 

Enhanced neuroplasticity might be underlying the improvements seen. Colcombe and colleagues demonstrated that older adults without dementia who performed aerobic exercises had greater grey and white matter volumes compared to adults who engaged in stretching and toning exercises [38]. Exercise has also been associated with functional connectivity between brain networks often affected by age, such as the default mode, frontal parietal, and frontal executive networks, in older adults without dementia [39]. While randomized controlled trials in AD patients examining the relationship between neuroplasticity and exercise are underway, correlational studies examining brain volumes and cardiorespiratory fitness have been done. In AD patients, cardiorespiratory fitness has been associated with brain volume. VO_2_^peak^, peak oxygen consumption, has been positively correlated with greater whole brain volume and white matter volume [40], notably in the inferior parietal lobule, hippocampal, and parahippocampal regions [41]. Future results of randomized controlled trials will improve our knowledge in this field of research. 

Overall, physical activity offers promising outcomes for cognition and physical health in the elderly population and AD patients.

## 4. Intellectual Activity 

Engagement in intellectually stimulating activities has been linked with reduced risk of developing AD and intellectual stimulation has been widely explored as a nonpharmacological treatment option for dementia [42]. 

Among cognitively normal older persons, randomized control trials employing intellectual training concluded that cognitive interventions produce protective and potentially long lasting positive effects in various cognitive domains as well as activities of daily living [43]. There is also evidence that frequent engagement in hobbies, including reading, puzzles, and games, for at least six hours per week reduces the risk of incident dementia [44]. The concept of intellectual stimulation as a preventative measure for dementia in healthy older adults can be parallel to the notion of building a “compensatory mechanism” or “cognitive reserve” [45–48].

Cognitive reserve refers to the hypothesis that individual differences shaped by inherent characteristics and external sources including intelligence, years of education, occupation, and intellectual activities, may provide neural protective support against dementia [45–47]. It has been argued that these collective life experiences may contribute to building cognitive reserve and, thus, provide skills to compensate for AD pathology [45–47]. In other words, a greater cognitive reserve might delay the appearance of dementia despite the presence of neuropathology, after which a rapid progression of cognitive decline may ensue once pathology is significant enough to result in AD diagnosis. Thus, AD patients with higher education and occupation accomplishments suffer more rapid decline in cognitive abilities when compared to AD patients with less education and occupational attainment following diagnosis [49]. Another study by Helzner and colleagues [50] investigated the relationship between premorbid leisure activity and rate of cognitive decline in AD patients. Leisure activities were classified into four categories: intellectual, social, physical, and other. Higher-frequency participation in intellectual leisure activities prior to AD diagnosis was associated with delayed AD onset followed by faster cognitive decline. The study by Helzner and colleagues [50] provides evidence for the benefits of intellectual stimulation on slowing down AD development. 

Besides reducing the risk of dementia, cognitive interventions later in life may affect functional decline in AD. Treiber and colleagues [51] explored the association between engaging in cognitively stimulating activities in late life and the rate of cognitive decline in incident AD. This study included a wide range of intellectual activities that required varying levels of cognitive demand, for example, completing puzzles, reading, watching television, listening to music, and cooking. The results suggested that higher-frequency participation in stimulating activities in early stages of dementia resulted in slower cognitive decline. However, as time progressed there was an overall decrease in participation in activities, which might reflect the nature of AD in terms of functional abilities. 

Intellectual stimulation can be divided into several categories including cognitive stimulation, cognitive training, and cognitive rehabilitation (Table 2) [48, 52–54]. Each type of stimulation targets various cognitive domains including memory and attention, or even more generally, activities of daily living [55]. The focus of this paper is to investigate the literature on the link between intellectual stimulation and rate of cognitive decline in patients with dementia. 

### 4.1. Cognitive Stimulation and Reality Orientation in Dementia

Cognitive stimulation therapy (CS) encompasses a variety of mentally stimulating activities and is less formal than other forms of cognitive training [53]. In addition, cognitive stimulation can be passive, such as listening to music, or active, such as playing a game [53]. The goal of CS is to enhance a wide range of functioning, including social skills, often through group discussions [48, 56]. An earlier randomized control trial included a total of 167 participants (97 in the treatment group and 70 in the control group). This study examined the relationship between CS, cognitive functioning, and quality of life [57]. The CS group participated in activities including crafts, games, and singing compared to a control group who received no intervention. The sessions ran for seven weeks and consisted of a total of 14 sessions, one 45-minute session twice a week. Assessment measures included cognitive tests and measures of quality of life, communication, behavior, global functioning, depression, and anxiety. The results indicated positive effects of CS on cognitive functioning as well as ADLs. Other studies found CS to be beneficial on measures of language [58], emotional wellbeing [58], and cognitive tests [57]. Unfortunately, not all studies found significant benefits of CS on dementia patients [59].

Reality orientation (RO) is a component of CS and focuses on orienting the individual with AD to person, place and time [52, 56]. Studies on RO in dementia indicate slight advantages on cognitive tests [60, 61]. A recent Cochrane review of fifteen randomized control trials investigated the effectiveness of RO and some CS in AD [56]. The review included 718 participants, with 407 participants in the treatment group and 311 in the control group. Intervention length varied between four weeks and 24 months, with sessions ranging from 30 to 90 minutes. Overall cognitive functioning was improved in AD patients receiving RO and CS, with some studies indicating additional positive changes to social and communicative functioning and quality of life [56]. 

Based on the current literature on the efficacy of CS and RO on decline in AD, it appears that both forms of intervention may provide encouraging results for slowing decline and improving quality of life [52, 56]. 

### 4.2. Cognitive Training and Cognitive Rehabilitation in Dementia

Cognitive training (CT) targets individual or multiple cognitive domains through teaching specific skills. It aims to improve functioning in the targeted area as well as the possibility of extending the improvements to more general functioning and other cognitive domains [54, 62]. Similarly, cognitive rehabilitation (CR) aims at improving daily functioning by identifying individualized strengths and weaknesses [56, 63]. Due to the significant amount of overlap between CT and CR, both interventions are sometimes combined [62].

Several reviews on CT suggest mixed outcomes in terms of effectiveness as a treatment option for AD [52, 62]. While several studies show improvement in specific cognitive domains targeted by training [64–66], others fail to report any significant changes [67] or extend results to more general functioning [65]. A Cochrane review by Clare and Woods [62] concluded that CT and CR provided no positive or negative effects on cognitive performance in AD compared to control groups. Conversely, Small and colleagues [68] found CT to have a negative impact on mood in patients with dementia and in their caregivers. A meta-analysis showed that CT had modest positive effects on various aspects of cognition, including learning, memory, executive and general functioning, and depression [55]. A randomized control trial using CR alone found that goal performance and satisfaction was improved compared to the control groups receiving relaxation therapy [63]. 

Another review investigated the literature on CT in preventing incident AD, preventing MCI conversion to AD, and slowing AD progression [48, 69]. The results suggested that CT may be most beneficial to healthy elderly and at risk individuals with MCI as opposed to AD treatment [48]. 

There is a lack of a consensus in the literature as to whether CT can ameliorate symptoms of AD [52, 62]. Although some studies show promising results for CT [55], others reveal improvement only in specific areas of cognitive training, such as learning personal information and aspects of attention [65]. CR, on the other hand, might produce more general benefits to cognitive functioning [63]. 

### 4.3. Specific Cognitive Domain Training in Dementia

Targets of intellectual stimulation can also be divided into unidomain and multidomain. While most cognitive interventions target multidomain areas of cognition and functioning, some are specific to memory training. Although some studies suggest memory rehabilitation can improve specific memory function with sufficient time and caregiver involvement [70], a randomized control study focusing on mnemonic memorization strategies found no significant difference between the treatment and control groups [71]. 

### 4.4. Comparing Interventions

Ballard and colleagues recently reviewed CT, CS, CR, and memory aids and concluded that all forms of intervention may offer modest effects, with most benefit attributed to CS [52]. In comparing CT and CS, both resulted in improvement of activities of daily living; however, only CS resulted in improvement in behavior [53, 72]. There are several reasons to explain differences in effectiveness of treatment types. There can be a significant overlap between types of intellectual stimulation, making it difficult to identify which types of interventions are most effective. The fact that some studies implementing only memory training were included in reviews of cognitive training [55] might also contribute to mixed findings in reviews of CT and cloud the effectiveness of each type of treatment. Moreover, differences in the past history and disease stages of the patients might also explain different results. Diversity in methodologies, such as sample size, variations in inclusion criteria, inconsistent outcome measures and protocols, also renders between study comparisons problematic [55]. In addition, not all studies control for medications such as cholinesterase inhibitors, which might contribute to positive effects along with stimulating activities [53].

Nonetheless, intellectual stimulation is a promising tool for slowing the progression of AD. More randomized control trials that compare generalizability of training programs, long-term effects, and rate of cognitive decline across different intervention types are needed to assess the most effective form of intellectual stimulation. 

## 5. Socialization

AD patients' feelings of incompetence, along with societal misconceptions, may accelerate the progression of AD [73]. So far, the relevance of the social environment surrounding an individual with AD has been mostly overlooked, but the results of increased social engagement on risk of dementia open the opportunity to incorporate socialization therapies in AD. 

### 5.1. Influence of Increased Socialization on Incident Dementia

The cognitive reserve hypothesis, as previously described [45–47], includes social engagement as an important skill set for delaying the development of AD. An increase in social engagement with the surrounding environment can be correlated with angiogenesis, synaptogenesis, and neurogenesis [74]. Fratiglioni et al. also explained the relevance of stress reduction in cognition through the stress hypothesis [74] and increased social engagement is associated with improved self-esteem [73]. The importance of social engagement in reducing the risk of AD can be further supported by the vascular hypothesis. It is important to note that vascular risk factors, which could be reduced with increased social engagement, can contribute to the pathogenesis and progression of AD [74, 75].

Table 3 showcases the importance of social engagement on the prevention of AD, there is a lack of research on the effects of social engagement on AD prognosis. Collaborative research in the fields of sociology, psychology, neuroscience, and medicine will be important in this area.

### 5.2. Influence of Socialization on AD Population

Many AD patients sense that their cognitive impairment isolates them from other people leading to anxiety, depression, societal withdrawal, and decreased self-confidence. Manipulating the social environment around AD patients may help them regain a sense of self-worth and a better attitude towards life. This may improve eating and exercise habits and social interactions, which may result in improved AD prognosis [73]. 

Shaping how cognitively normal individuals relate to AD patients can play an important role in improving patients' social environment and interactions [73]. It is extremely important that surrounding healthy individuals (friends, family members, etc.) maintain a supportive atmosphere towards AD patients to ensure that, regardless of their cognitive abilities, they have a place in society and that their identity is valued. After multiple experiences, MacRae [73] reported that married AD patients whose spouses encouraged them to live life as normally as possible, and to remain socially engaged, preserved a “positive sense of self while living with dementia.” This led to healthier eating habits and increased physical exercise and participation in cognitively and socially stimulating activities, thereby improving quality of life [73]. 

In further support of how a positive social atmosphere can benefit individuals with AD, Keller et al. [81] analyzed how shared mealtimes can enhance social engagement. With disease progression, alterations must be made to accommodate for changes in eating conduct (difficulties with using utensils or wandering), swallowing ability, and the ability to prepare food safely. Therefore, an event that was once a traditional social occasion may become an event filled with tension, frustration, and disappointment. As a result, the social experience associated with mealtimes may become limited and possibly nonexistent. However, reports show that AD patients benefit from shared mealtimes by getting motivated through another's physical presence to improve their attentional, planning, and decision making abilities [81]. 

Loneliness is widely reported by patients with dementia, arising from the feeling of not having anyone to relate to, dissatisfaction with one's way of living, and social isolation. Recent studies have attempted to reduce this sense of “loneliness” and assess its effects on cognition [76]. Pitkala et al. [75] examined the effects of enhancing social stimulation on cognitive outcomes in the elderly and patients with mild dementia. Volunteers were divided into three social intervention groups, according to their preference: (1) art, inspiring activities, and discussions, (2) exercise and discussions, and (3) therapeutic writing and discussions. Within each group, they were further divided into intervention or placebo. Participants in the intervention groups improved significantly over the course of 3 months on the Alzheimer's Disease Assessment Scale (ADAS-Cog) compared to control groups. Furthermore, a health-related quality of life (HRQol) questionnaire conducted at baseline and at 12 months revealed significant improvements in quality of life in the social intervention groups [75]. 

Support groups are another intervention aimed to assist with the psychosocial difficulties that patients diagnosed with early-stage dementia face [82]. A clinical trial compared quality of life (QOL), depression, and family communication outcomes between an early-stage memory loss (ESML) support group and a wait-list (WL) control group [83]. The ESML condition involved 90-minute weekly sessions for 9 weeks. The WL condition did not participate in the social support groups, but received educational material from their local Alzheimer's Association group and were encouraged to contact relevant support services should any questions or concerns arise. Results showed that in comparison to control participants, ESML participants expressed better QOL and less depressive symptoms after 9 weeks. Furthermore, the improved QOL among ESML participants was associated with an improvement of cognition and family communication [83]. 

Results have shown that although caregivers are willing to help their loved ones; at times at a personal cost, AD caregivers have a much higher chance of being clinically depressed than age-matched controls [84, 85]. Thus, many psychosocial therapies have been explored to primarily help caregivers, which frequently lead to better care of patients. Mittelman explains that individualized counseling and involvement in caregiver support groups result in long-lasting lower caregiver distress, even at followups at one and three years later [86]. Furthermore, lower distress and depressive symptoms were observed across caregivers for patients with varying dementia severity, living conditions, and after death of the patient [86]. 

It appears that enhanced social interactions may decrease the risk of AD and improve patients' QOL, self-confidence, and possibly prognosis. Although it appears that socialization is important in supporting cognitive abilities in the normal elderly, further research is needed to clarify how and to what extent socialization benefits patients with AD.

## 6. Multimodal Therapy 

Given that physical exercise, intellectual stimulation, and socialization have individually shown to benefit AD patients, we summarize the results of multimodal interventions, combining at least two of the three activity types. 

Coelho et al. [87] found that participants who engaged in an exercise program while simultaneously completing cognitive tasks had significantly higher scores (*P* < 0.001) than the control group on the Frontal Assessment Battery, which measures frontal lobe cognitive function. Furthermore, participants in the control group demonstrated decreased executive and visuospatial abilities through lower scores on a clock drawing test. The physical exercise program consisted of aerobic, flexibility and balance exercises, and resistance training. Cognitive tasks included generating words according to specific criteria (e.g., flowers, animals) or reacting to sensory stimuli such as verbal commands. These findings suggest that there is a place for physical exercise combined with intellectual stimulation in AD management. 

Arkin [88] provides further support for the use of multimodal intervention through a study of 24 AD patients engaged in exercise (aerobic and weight training), cognitive tasks (taking place before, during, and after exercise), and social activities. Compared to controls, the 4-year intervention group experienced no decline in several cognitive measures: MMSE, Clinical Dementia Rating Scale, verbal fluency, Boston Naming, and WAIS-R Comprehension. Burgener et al. [89] conducted a trial in which 24 early-AD patients received a multimodal intervention (exercise, cognitive behavioral therapy, and support group) and were compared to 19 patient control group who received attention-control educational programs. In addition to physical and behavioral outcome benefits, there was a significant difference in MMSE scores between the intervention and control groups, not present at baseline. As such, both studies suggest that multimodal intervention programs can help delay the cognitive decline characteristic of AD.

It is important to note, however, that evidence supporting multimodal intervention is not yet conclusive. A pilot study of 14 AD patients by Maci et al. [90] found that participants engaged in a multimodal program of exercise (balance, gait, and coordination), cognitive stimulation, and socialization (talking/singing with other participants) led to reduced depression and improved anxiety and quality of life. However, there were no significant between-group differences in cognition as measured by the MMSE and Frontal Assessment Battery. It is possible that behavioral improvements may have been secondary to the very small sample size. Conflicting findings such as these indicate the need for further research in this area. 

While the impact of multimodal interventions (and perhaps even each intervention independently) on reducing cognitive decline in AD remains unclear, it appears that patients may benefit in other ways from physical exercise, intellectual stimulation, and socialization. More research into the effects of multimodal therapies is required.

## 7. Conclusions

In summary, this paper highlights key research on the effectiveness of nonpharmacological treatment options, specifically exercise, intellectual stimulation, and socialization, on the progression of AD. Based on our findings in the literature, all three intervention strategies may provide positive effects on cognition and overall wellbeing. More specifically, physical exercise appears to benefit various aspects of cognition in AD patients including stabilization of MMSE scores, and improved attention, memory and recognition, and ability to perform ADLs. It has also been demonstrated that regular exercise is helpful in reducing the risk of AD development and progression. Future studies should include a broader range of exercises to determine the most effective exercise regime for AD patients. Increased social interaction has been shown to benefit Alzheimer's patients by minimizing one's sense of loneliness, isolation, stress, and vascular factors that contribute to cognitive decline. It can also improve patients' sense of self-worth. However, limited studies exist on the benefits of socialization on cognition in the AD population. Studies focusing on these areas would help verify if a social component would provide more than simply behavioral benefits. In addition, intellectual stimulation may benefit AD patients by improving their cognition, emotional and social wellbeing as well as performance of ADLs. However, inconsistent results on the effectiveness of intellectual stimulation in delaying cognitive decline in AD patients demonstrate the need for further research. 

It is necessary for future research to identify and address limitations found in the current literature. Patient recruitment can be a significant challenge in those with moderate-to-advanced AD due to the necessary comprehension levels for study completion. In addition, as dementia progresses, patients may become increasingly tired and frustrated, leading to higher attrition rates. Some of the studies do not provide adequate characterization of the enrolled population, rendering comparisons difficult. The ability to make between-study comparisons is further limited by the broad range of physical, cognitive, and social activities used in nonpharmacological therapies. Determining which nonpharmacological therapy is the most beneficial to the AD population is difficult due to the widespread and overlapping positive effects of each activity. As the goal of this paper was to provide a general overview of nonpharmacological interventions in AD, we were limited in our ability to produce statistical analysis. Therefore, a meta-analysis is suggested in order to obtain quantitative results. 

Based on the literature presented in this paper, patients experience less depression and improved quality of life, ability to perform ADLs, and social relationships. Most importantly, interventions that combine physical exercise and socialization with cognitive stimulation are often highly enjoyable for patients, leading to participants expressing their approval by saying: “This has been the only thing I have found that feels like I am doing something to help myself” [89]. Thus, the aforementioned nonpharmacological therapies are recommended in AD management. 

## Figures and Tables

**Table 1:** Summary of randomized controlled trials of physical exercise regimes in AD patients.

Study	Length and frequency	Type of exercise	Sample	MMSE (baseline mean)	Outcome measures	Major findings
Kemoun et al. [27]	15 wks; 1 h sessions 3x/wk	Aerobic, balance, and endurance	*n*_ex_ = 16 *n*_*c*_ = 15	12.6	Walking speed, stride length, double limb support time, and ERFC	Higher ERFC and walking parameter scores in exercise group; positive correlation between walking parameters and ERFC scores

Miu et al. [28]	3 mos; ~1 h sessions 2x/wk	Aerobic, flexibility	*n*_ex_ = 36 *n*_*c*_ = 49	20 (median)	SF-12, 6 min walk, functional reach, BBS, MMSE, ADAS-Cog, and CSDD	Improvement in walking and functional reach in exercise group

Roach et al. [29]	16 wks; 15–30 min sessions 5x/wk	Strength, flexibility, endurance, and balance	*n*_ex_ = 28 *n*_soc_ = 25 *n*_walk_ = 29	10.7	ACIF, 6 min walk, and MMSE	Improved transfer in exercise group

Rolland et al. [30]	1 yr; 1 h session 2x/wk	Aerobic, strength, flexibility, and balance	*n*_ex_ = 67 *n*_*c*_ = 67	8.8	Katz Index of ADLs, walking speed, get-up-and-go, one-leg balance, MNA, NPI, and MADRS	Less decline in overall ADLs in exercise group

Steinberg et al. [31]	12 wks; ~3x/wk	Aerobic, strength, balance, and flexibility	*n*_ex_ = 14 *n*_*c*_ = 13	20.1	YPAS, 8-ft walk, JTT, sit-to-stand, MMSE, BNT, HVLT, ADQRL, NPI, CSDD, and SCB	Improved performance in JTT and sit-to-stand, increased depression, and decreased ADQRL in exercise group

Venturelli et al. [32]	24 wks; 30 min sessions 4x/wk	Mobility, aerobic	*n*_ex_ = 12 *n*_*c*_ = 12	13	6 min walk, Barthel Index of ADLs, and MMSE	Improvements in walking and ADLs, slower decline in MMSE in exercise group

Vreugdenhil et al. [33]	4 mos; >30 mins daily	Strength, balance, and aerobic	*n*_ex_ = 20 *n*_*c*_ = 20	22.9	ADAS-Cog, MMSE, functional reach, Timed Up and Go, sit-to-stand, Barthel Index of ADLs, GDS, CIBIC-plus, and Zarit Burden Interview	Improved scores on ADAS-Cog, MMSE, CIBIC-plus, functional reach, Timed Up and Go, sit-to-stand, and ADLs in exercise group

Y$\overset{`}{\text{a}}$g$\ddot{\text{u}}$ez et al. [34]	6 wks; 1.5 h sessions 1x/wk	Flexibility, eye-hand coordination	*n*_ex_ = 15 *n*_*c*_ = 12	22.1	CANTAB-Expedio	Improvements in attention, visual memory recognition, and working memory in exercise group

*n*_ex_, *n*_*c*_, *n*_soc_, *n*_walk_: number of participants in exercise program, control group, social conversation group, and walking group, respectively. ACIF: Acute Care Index of Function, ADAS-Cog: Alzheimer's Disease Assessment Scale-Cognitive Subscale, ADL: activities of daily living, ADQRL: Alzheimer's Disease Quality Related Life Scale, BBS: Berg Balance Scale, BNT: Boston Naming Test, CANTAB-Expedio: The Cambridge Neuropsychological Test Automated Battery, CIBIC-plus: Clinician's Interview-Based Impression of Change Plus Caregiver Input, CSDD: Cornell Scale for Depression in Dementia, ERFC: Rapid Evaluation of Cognitive Function, GDS: Geriatric Depression dcale, HDS: Hamilton Depression Scale, HVLT: Hopkins Verbal Learning Test, JTT: Jebsen Total Time, MADRS: Montgomery-Asberg Depression Rating Scale, MMSE: Mini-Mental State Examination, MNA: Mini-Nutritional Assessment, NPI: Neuropsychiatric Inventory, SCB: Screen for Caregiver Burden, SF-12: SF-12 Quality of Life Questionnaire, SF-36: Short-Form Health Survey, SIP: Sickness Impact Profile, and YPAS: Yale Physical Activity Survey.

**Table 2:** Summary of trials involving cognitive interventions in AD patients.

Study	Length and frequency	Type of stimulation	Sample	MMSE (baseline mean)	Outcome measures	Major findings
Buschert et al. [59]	6 months; 2 hours 1x/wk	Multicomponent intervention with focus on CS	n_ex_ = 8 *n*_*c*_ = 7	Experimental: 24.5 Control: 25.3	(i) MMSE (ii) ADAS-Cog (iii) TMT (iv) RBANS story memory and recall (v) QoL-AD (vi) MADRS	(i) No significant benefit

Chapman et al. [58]	8 wks; 90-minute sessions 1x/wk	Cognitive stimulation	*n*_ex_ = 28 *n*_*c*_ = 26	20.87	(i) MMSE (ii) ADAS-Cog (iii) TFLS NPI-irritability and apathy (iv) QoL-AD (v) CBIC	(i) Modest positive effects on language, global and functional abilities, and emotional wellbeing in experimental group compared to control group just treated with donepezil

Spector et al. [57]	7 wks; 45-minute sessions 2x/wk	Cognitive stimulation	n_ex_ = 97 n_c_ = 70	Experimental: 14.2 Control: 14.8	(i) MMSE (ii) ADAS-Cog (iii) QoL-AD (iv) HCS (v) Cornell Scale for Depression in Dementia (vi) CAPE-BRS (vii) CDR	(i) Significant improvement on the MMSE, ADAS-COG, and QoL-AD

Onder et al. [60]	25 wks; 30-minute sessions 3x/wk	Reality orientation	*n*_ex_ = 70 *n*_*c*_ = 67	Experimental: 20.2 Control: 19.9	(i) MMSE (ii) ADAS-Cog (iii) Functional status: Barthel Index, (iv) IADL (v) NPI (vi) Family caregiver outcomes: HRSD, HRSA, Caregiver Burden Inventory, SF-36	(i) Small improvement in MMSE and ADAS-COG

Loewenstein et al. [64]	12–16 weeks; 45-minute sessions 2x/wk	Cognitive rehabilitation/cognitive training	n_ex_ = 25 *n*_*c*_ = 19	Experimental: 23.40 Control: 24.53	(i) The face-name association task: (ii) Orientation (iii) The continuous performance test (iv) Procedural object-memory evaluation (v) Modified Making-Change-For-A-Purchase Task (vi) Balancing-A-Checkbook Task (vii) RMBPC (viii) B-ADLS (ix) CES-D (x) IQCODE	(i) Training specific improvement in orientation, face-name recall, processing speed, and making change compared to control group receiving mental stimulation

Davis et al. [65]	5 weeks; 1-hour sessions 1x/wk; home practice 30 minutes per day 6x/wk	Cognitive training	*n*_ex_ = 19 *n*_*c*_ = 18	Experimental: 21.84 Control: 22.78	(i) MMSE (ii) WMS-R logical memory (iii) WMS-R visual reproduction (iv) WAIS-R digit span (v) VSAT (vi) Controlled oral word association test (vii) Category fluency (viii) Finger taping test (ix) GDS (x) Quality of life patient	(i) Improvement in attention, recall of personal information, and face-name recall compared to mock control group (ii) Results did not extend to other neuropsychological tests or quality of life

Koltai et al. [67]	Group program: 5 wks; 1-hour sessions 1x/wk Individual program: 6 wks; 1-hour sessions 1x/wk with caregiver involvement in last 10–15 minutes	Cognitive rehabilitation/cognitive training	*n*_ex_ = 14 *n*_*c*_ = 8	Experimental: 22.9 Control: 26.6	(i) GDS and relative GDS (ii) EMQ and relative EMQ (iii) CERAD (iv) MMSE	(i) No significant differences between the experimental group and the wait-list control group (ii) Participants with insight about self-deficits perceived greater gains in memory compared to participants with no insight

Quayhagen et al. [66]	8 weeks; 1-hour sessions 5x/wk	Cognitive training	*n*_AD_ = 103 *n*_*c*_ = 103 (i) Numbers in each group (cognitive training, dyadic counseling, dual supportive seminar, and early-stage day care) not specified	Not reported	(i) Immediate memory composite: WMS-R Logical Memory 1 and visual reproduction 1, DRS (memory) (ii) Delayed memory composite: WMS logical memory 2, visual reproduction 2 (iii) Verbal fluency composite: controlled oral word association, DRS (initiation) (iv) Problem solving composite: geriatric coping schedule, DRS (conceptualization) (v) Memory and Behavior Problems Checklist (part A)	(i) Improvement in memory and communication with caregiver (ii) Other types of interventions (dual support seminar, early-stage daycare) had positive effects on communication and interactions

Cahn-Weiner et al. [71]	6 wks; 45-minute sessions 2x/wk	Memory training	*n*_ex_ = 17 *n*_*c*_ = 17	Experimental: 24.3 Control: 25.1	(i) HVLT-R (ii) BVMT-R (iii) BNT (iv) COWA (v) JLO (vi) TMT (Parts A and B) (vii) ADL questionnaire (viii) EMQ	(i) No significant effects

*n*_ex_, *n*_*c*_, *n*_AD_: number of participants in intellectual stimulation program, control group, and group diagnosed with AD. MMSE: Mini-Mental state examination, ADAS-Cog: Alzheimer's Disease Assessment Scale-Cognitive Subscale, TMT: Trail Making Test, RBANS: Repeatable Battery for the Assessment of Neuropsychological Status Versions A (before testing) and B (after testing), QoL-AD: quality of life-Alzheimer's disease, MADRS: Montgomery-Asberg Depression Rating Scale, TFLS: Texas Functional Living Scale, NPI: Neuropsychiatric Inventory, CIBIC: Clinician's Interview-Based Impression of Change, HCS: Holden Communication Scale, CAPE-BRS: The Clifton Assessment Procedures for the Elderly-Behaviour Rating Scale, CDR: The Clinical Dementia Rating Scale, IADL: instrumental activities of daily living, HRSD: Hamilton Rating Scales for Depression, HRSA: Hamilton Rating Scales for Anxiety, SF-36: The Medical Outcomes Study 36-Item Short-Form General Health Survey, RMBPC: The Revised Memory and Behavior Problems Checklist, B-ADLS: The Bayer Activities of Daily Living Scale, CES-D: The Center for Epidemiological Studies-Depression Scale, IQCODE: the informant questionnaire of the cognitive decline in the elderly scale, WMS-R: Wechsler Memory Scale-Revised, WAIS-R: Wechsler Adult Intelligence Scale-Revised, VSAT: The Verbal Series Attention Test, GDS: Geriatric Depression Scale, EMQ: Everyday Memory Questionnaire, CERAD: Consortium to Establish a Registry for Alzheimer's Disease Test Battery, DRS: The Mattis Dementia Rating Scale, HVLT-R: Hopkins Verbal Learning Test Revised, BVMT-R: Brief Visual Spatial Memory Test Revised, BNT: Boston Naming Test, COWA: Controlled Oral Word Association Test, and JLO: Judgment of Line Orientation.

**Table 3:** Summary of studies on the effect of socialization on incident dementia.

Study	Length and frequency	Protocol	Sample	MMSE (baseline mean)	Outcome measures	Major findings
Wilson et al. [76]	4 years; annual check up	Longitudinal clinicopathological cohort study	*n* = 823		(i) Clinical diagnosis of AD (ii) Change in measures of global cognition and specific cognitive functions	(i) Loneliness associated with cognitive decline and development of AD

Wang et al. [77]	Data collection (9 years)	Longitudinal population-based study	*n* = 776	27.3	(i) Frequency of social and leisure activities engaged 6.4 years before diagnosis (ii) Baseline MMSE (iii) MMSE of incident dementia cases	(i) Socially and mentally stimulating activity may preserve mental functioning in the elderly, reducing risk of dementia

Friedland et al. [78]		Questionnaire data collection	*n*_AD_ = 193 *n*_HC_ = 358		(i) Monthly involvement in possible 26 nonoccupational activities at early adulthood and middle adulthood	(i) AD patients are less active in midlife than HC participants

Bennet et al. [79]	6-7 years	Longitudinal, epidemiological clinicopathological cohort study	n = 89	25.8 (not used in analysis)	(i) Annual clinical evaluation (ii) Brain autopsy at death (iii) Social network size (number of individuals seen at least once/month)	(i) Larger social network sizes observed in participants with higher level of cognition

Fratiglioni et al. [80]	3 years	Longitudinal community-based study	*n*_HC_ = 1203	>23	(i) Social network at baseline, clinical evaluation at baseline and 3 years	(i) Limited social network ties and interaction increased risk of developing dementia

*n*_HC_, *n*_AD_: number of participants in healthy control group and group diagnosed with AD. MMSE: Mini-Mental State Examination.
